# Firearm screening in pediatric patients

**DOI:** 10.3389/fped.2024.1415612

**Published:** 2024-06-21

**Authors:** Aarani Kandeepan, Jessica Lee, Dayanand Bagdure, Nan Garber, Jenni Day, Adrian Holloway, Richard Lichenstein, Joseph Slattery, Alexa Wolfe, Jenna Wadsworth, Julianne Moss, Nicole Davie, Cortney Foster

**Affiliations:** ^1^ Department of Pediatrics, University of Maryland, Baltimore, MD, United States; ^2^ Department of Pediatrics, The Children’s Hospital of Montefiore, New York, NY, United States; ^3^ Department of Pediatrics, Ochsner LSU Health, Shreveport, LA, United States; ^4^ Department of Pediatrics, West Virginia University, Morgantown, WV, United States; ^5^ Department of Nursing, University of Maryland, Baltimore, MD, United States; ^6^ Department of Pediatrics, St. Josephs Hospital, Baltimore, MD, United States; ^7^ Department of Pediatrics, University of Wisconsin, Madison, WI, United States

**Keywords:** firearm, firearm screening, pediatrics, firearm safety, firearm injury

## Abstract

**Introduction:**

In the United States, firearm-related injuries are the leading cause of death among children and adolescents 1–19 years of age. Although many pediatricians believe addressing firearm safety is important and have guidance from organizations like the American Academy of Pediatrics, few routinely screen and counsel on firearm safety. The goal of this project was to screen all patients presenting to the pediatric emergency department, pediatric floor, and pediatric intensive care unit for the presence of firearms in the home, firearm storage practices, and whether they had previously received any firearm counseling by medical professionals.

**Methods:**

A 13-item survey was administered to each participant. Items included demographic information, willingness to answer questions about firearms, practice of asking questions about firearms, previous counseling from medical professionals about the presence of firearms in the home and the presence of firearm in their personal home as well as storage practices.

**Results:**

A total of 200 parents responded to the survey. Of those that responded to the survey, 171 (85.5%) did not have a firearm in the home and 28 (14%) did have a firearm in the home. 75% (*n* = 21) had never had a medical provider discuss firearm safety with them. 100% had never been asked by another parent about the presence of a firearm in their home when a child came over for a playdate. 39% (*n* = 11) of parents with a firearm in the home had asked other parents whether they have a firearm in the home where their child goes to play.

**Discussion:**

Findings from our study highlight a significant lack of screening of our pediatric patients both in the inpatient and outpatient settings, with the majority reporting that they had never been asked by a medical provider about firearm safety. In addition, three quarters of parents with a firearm in the home reported that they did not mind answering questions about firearms yet none had been asked by other parents about firearms. Thus, although firearm possession and safety is considered to be a sensitive topic, many parents are willing to discuss it with their health care providers and other parents.

## 1 Introduction

In the United States, firearm-related injuries are the leading cause of death among children and adolescents 1–19 years of age. Although prior analyses through 2016 showed motor vehicle accidents as the leading cause of death in this age group, firearm-related injuries surpassed motor vehicle accidents as the leading cause of death in 2020 (1). While it was thought that this increase may have been related to the fears and anxiety related to the onset of the COVID-19 pandemic, this trend continued in 2021. In 2021, 4,750 children and adolescents died from firearms. When comparing 2018–2021, there was a 41.6% increase in the firearm-related death rate. Thus, firearms are a significant source of pediatric mortality now more than ever (2). Not only do firearm injuries lead to mortality, but they can also result in lasting disability, adverse impacts on the mental health of children and their families, as well as high use of medical resources. Compared to other high-income countries, children in the United States are uniquely affected by this problem. This is partly due to the presence of firearms in the home. It is estimated that about 4.6 million children live in homes with at least one firearm that is stored loaded and unlocked (3). Firearms stored at home are often accessible by children and adolescents and can be used in accidental shooting incidents among children, youth suicides and school shootings (4).

Unintentional firearm injuries are especially a concern among younger children. Most firearm injury hospitalizations for children under 15 years-old are due to unintentional injuries (5). A 2017 study found most children who died from unintentional firearm injuries had died in the context of playing with a firearm or showing it to others, and they were most often shot by another child in a similar age range (6). This is in part because most young kids will touch a firearm if they find one and will do so, even if they are told not to (7, 8). Parents often do not realize how interested their child may be in firearms and are poor predictors of how their child will act around firearms (7). Furthermore, many parents believe their child does not know the location of their firearm and that their child has never handled a firearm, despite their child stating otherwise. Baxley et al. found that nearly 3 out of 4 children under 10-years-old said they knew the location of their parent's firearm and many of the children had handled firearms without their parent's knowledge (9). For adolescents, it is estimated that even the presence of a firearm in the home increases that adolescent's risk of suicide by up to four-fold (10).

A proven method to prevent pediatric firearm injuries and fatalities is through promoting safe firearm storage, which includes storing firearms locked and unloaded and maintaining the ammunition locked and stored in a separate location (4). The American Academy of Pediatrics (AAP) Bright Futures guidelines recommend that pediatricians counsel parents on firearm safety as a part of routine care. They recommend asking parents if there are firearms in the home and how those firearms and ammunition are stored as well as instructing parents to ask others if there are firearms in the homes where their children play (11–13). Many pediatricians agree that firearm safety is important, and it is beneficial to address with their patients (14–19). Studies evaluating the effectiveness of firearm safety counseling have found that counseling can improve firearm storage behaviors, especially when combined with written handouts, motivational interviewing techniques, and free firearm safety devices (20–24).

Although many pediatricians believe addressing firearm safety is important and have guidance from organizations like the American Academy of Pediatrics, few routinely screen and counsel on firearm safety (14–18, 24–38). If pediatricians do screen, they usually do so for patients at greater risk of firearm injury, such as adolescents, rather than conducting universal screening (33). Moreover, firearm safety screening and counseling most often occurs in the outpatient setting, rather than inpatient setting. In 2020, Monroe et al. found that the firearm screening rate among pediatricians at their tertiary-care pediatric hospital was very low at 0.4% (39). While most pediatricians agree they should address the issue, many do not because they lack the time, lack the appropriate training, are unsure it will be effective, and are reluctant to address the issue due to legal, political and other reasons (15, 16, 18–31, 40–45). In a similar study Oddo et al. looked at inpatient firearm screening within a tertiary hospital setting. They demonstrated that firearm screening increased from 0.2% to 39% within a 25-month period of intentional effort. However, similar barriers were reported, including lack of time and feeling that it was not an appropriate setting (46).

Despite these barriers, studies have found that although opinions may vary, the majority of parents are willing to discuss firearm safety with their pediatrician (37, 47–49). One study found that having a professional talk to parents about firearm safety can be helpful in opening the discussion since both parents may not agree on firearm safety as well as other child-raising topics (45). Additionally, even if parents do not agree with the physician's advice, parents said it could help them “think it over” (50). While adolescents may be less willing to talk to their pediatricians about firearm safety, one study found that if their physician asked them about firearms, most adolescents would be willing to discuss it (51). In addition, a study found that with some of the recent firearm-related incidents in the national spotlight, such as mass shootings, there has been an increase in public awareness about the importance of firearm safety, and thus it has become easier for pediatricians to start these conversations (44).

Thus, while pediatricians may be hesitant, it is critical that they talk about firearm safety with their patients and their parents. Inpatient pediatric providers have an opportunity to provide firearm safety screening and counseling since they currently rarely do so (46). The inpatient settings has other advantages as well, as the hospital context provides more resources and providers and there are more caregivers present, thereby allowing these parties to have these important conversations (39). In addition, hospital pediatric providers are already well versed in safety discussions about tobacco cessation and car seats, and the topic of firearm safety should be treated in the same way (52). Especially at academic hospitals, pediatric providers can teach trainees about the importance of firearm safety screening and counseling, which can have a lasting impact on the future pediatric workforce (52). The goal of this project was to screen all patients presenting to the pediatric emergency department, pediatric floor, and pediatric intensive care unit for the presence of firearms in the home, firearm storage practices, and whether they had previously received any firearm counseling by medical professionals.

## 2 Methods

The protocol was reviewed by the University of Maryland, Baltimore (UMB) Institutional Review Board (IRB) and was determined to be exempt under 45 CFR 46.101(b) from IRB review. Parents of children presenting to the pediatric emergency department, pediatric floor and pediatric intensive care unit of an urban tertiary care center with 16,000 annual pediatric emergency department visits and 1,000 annual pediatric intensive care unit admissions were screened for inclusion. Responders were included if they were parents aged 17 years or greater and willing to participate in a survey regarding firearms. A 13-item survey was administered to each participant. Items included demographic information, willingness to answer questions about firearms, practice of asking questions about firearms, previous counseling from medical professionals about the presence of firearms in the home and the presence of firearm in their personal home as well as storage practices (see Supplementary File S1: Firearm survey). Following completion of the firearm survey, parents were given a dual sided firearm safety handout detailing dangers of firearms and safe storage practices (see Supplementary File S2: Safety Handout). Data was collected over a two-year period.

### 2.1 Statistical analysis

Data were analyzed using SPSS-25. Descriptive statistics (frequency, percent) were calculated to describe the sample and to summarize responses to firearm survey questions. All variables included in the analysis were categorical. Data were evaluated for completeness and no participants or variables were missing more than 4% of data, excluding responses on whether firearm/ammunition was locked which was missing responses for 6.5% of participants. No data imputation method was required. A Chi-Square test was used to test for differences between providers discussing firearm safety (yes/no) and demographic variables (parent age, gender, race/ethnicity, number of children in the home, ages of youngest and oldest children in the home). Separate analysis was conducted with a Chi-Square test to test for differences between presence of a firearm in the home (yes/no) and demographic variables (parent age, gender, race/ethnicity, number of children in the home, ages of youngest and oldest children in the home). A Chi-Square test was also used to test for differences between presence of a firearm in the home (yes/no) and firearm survey questions.

## 3 Results

A total of 200 parents responded to the survey. Ages of responders ranged from 17 to 60 years old and 39% (*n* = 79) of our parental responders were 31–40 years old. We had 82% females (*n* = 165) and 16% males (*n* = 33). 59% of our parents were black (*n* = 118) and 29% were white (*n* = 59). The majority (85%) of our responders had 1–3 children in the home. 83% of our survey responders had children of age 10 years or less in the home.

Of the total of 200 survey respondents, 79% (*n* = 158) reported that no medical provider had ever spoken to them about firearm safety, and 96% reported that other parents had never asked them about the presence of a firearm in the home. No significant difference was found between whether a provider discussed firearm safety and demographic variables.

Of those that responded to the survey, 171 (85.5%) did not have a firearm in the home and 28 (14%) did have a firearm in the home. We found a significant association between race and the presence of firearms in the home. White parents were more likely than non- white parents to have a firearm in the home, *X^2^* (6, *n* = 192) = 12.971, *p* < .05.

Of the 28 parents that had a firearm at home, 75% (*n* = 21) responded that they did not mind answering questions about firearms. 75% (*n* = 21) had never had a medical provider discuss firearm safety with them. 100% had never been asked by another parent about the presence of a firearm in their home when a child came over for a playdate. 39% (*n* = 11) of parents with a firearm in the home had asked other parents whether they have a firearm in the home where their child goes to play. Of the 28 parents with a firearm in the home, 26 (93%) reported that the firearm and ammunition were locked (Table 1).

**Table 1:** Distribution of survey responses based on demographics and firearm ownership.

		Frequency	Percent	Valid percent	Cumulative percent
Age Range					
Valid	17–20	4	2.0	2.1	2.1
	21–30	62	31.0	32.3	34.4
	31–40	79	39.5	41.1	75.5
	41–50	36	18.0	18.8	94.3
	51–60	11	5.5	5.7	100.0
	Total	192	96.0	100.0	
Missing	999	8	4.0		
Total		200	100.0		
Gender					
Valid	Male	33	16.5	16.7	16.7
	Female	165	82.5	83.3	100.0
	Total	198	99.0	100.0	
Missing	999	2	1.0		
Total		200	100.0		
Race					
Valid	Black	118	59.0	61.5	61.5
	White	59	29.5	30.7	92.2
	Asian	5	2.5	2.6	94.8
	Caribbean	1	.5	.5	95.3
	Hispanic/Latino	4	2.0	2.1	97.4
	Mixed Race	4	2.0	2.1	99.5
	Other	1	.5	.5	100.0
	Total	192	96.0	100.0	
Missing	999	8	4.0		
Total		200	100.0		
Number of children in home					
Valid	0	2	1.0	1.0	1.0
	1	56	28.0	28.1	29.1
	2	71	35.5	35.7	64.8
	3	43	21.5	21.6	86.4
	4	15	7.5	7.5	94.0
	5	4	2.0	2.0	96.0
	6	7	3.5	3.5	99.5
	7	1	.5	.5	100.0
	Total	199	99.5	100.0	
Missing	999	1	.5		
Total		200	100.0		
Age of youngest child in home					
Valid	N/A	2	1.0	1.0	1.0
	<1 year	62	31.0	31.2	32.2
	1–5 years	72	36.0	36.2	68.3
	6–10 years	32	16.0	16.1	84.4
	11–15 years	18	9.0	9.0	93.5
	>15 years	13	6.5	6.5	100.0
	Total	199	99.5	100.0	
Missing	999	1	.5		
Total		200	100.0		
Age of oldest child in home					
Valid	N/A	59	29.5	29.6	29.6
	<1 year	2	1.0	1.0	30.7
	1–5 years	21	10.5	10.6	41.2
	6–10 years	37	18.5	18.6	59.8
	11–15 years	48	24.0	24.1	83.9
	>15 years	32	16.0	16.1	100.0
	Total	199	99.5	100.0	
Missing	999	1	.5		
Total		200	100.0		
Do you mind answering questions about guns?					
Valid	No	139	69.5	70.6	70.6
	Yes	58	29.0	29.4	100.0
	Total	197	98.5	100.0	
Missing	999	3	1.5		
Total		200	100.0		
Have other parents asked you about guns?					
Valid	No	193	96.5	96.5	96.5
	Yes	7	3.5	3.5	100.0
	Total	200	100.0	100.0	
Do you ask other parents about guns?					
Valid	No	142	71.0	72.1	72.1
	Yes	55	27.5	27.9	100.0
	Total	197	98.5	100.0	
Missing	999	3	1.5		
Total		200	100.0		
Has any medical provider talked to you about gun safety?					
Valid	No	158	79.0	79.0	79.0
	Yes	42	21.0	21.0	100.0
	Total	200	100.0	100.0	
Do you have a gun in the home?					
Valid	No	171	85.5	85.9	85.9
	Yes	28	14.0	14.1	100.0
	Total	199	99.5	100.0	
Missing	999	1	.5		
Total		200	100.0		
Is your gun/ammunition locked?					
Valid	Not Applicable	143	71.5	76.5	76.5
	No	14	7.0	7.5	84.0
	Yes	29	14.5	15.5	99.5
	Unsure	1	.5	.5	100.0
	Total	187	93.5	100.0	
Missing	999	13	6.5		
Total		200	100.0		
Age Range^b^					
Valid	17–20	4	2.3	2.5	2.5
	21–30	53	31.0	32.5	35.0
	31–40	66	38.6	40.5	75.5
	41–50	30	17.5	18.4	93.9
	51–60	10	5.8	6.1	100.0
	Total	163	95.3	100.0	
Missing	999	8	4.7		
Total		171	100.0		
Age Range^a^					
Valid	21–30	9	32.1	32.1	32.1
	31–40	12	42.9	42.9	75.0
	41–50	6	21.4	21.4	96.4
	51–60	1	3.6	3.6	100.0
	Total	28	100.0	100.0	
Gender^b^					
Valid	Male	26	15.2	15.4	15.4
	Female	143	83.6	84.6	100.0
	Total	169	98.8	100.0	
Missing	999	2	1.2		
Total		171	100.0		
Gender^a^					
Valid	Male	7	25.0	25.0	25.0
	Female	21	75.0	75.0	100.0
	Total	28	100.0	100.0	
Race^b^					
Valid	Black	107	62.6	64.8	64.8
	White	43	25.1	26.1	90.9
	Asian	5	2.9	3.0	93.9
	Caribbean	1	.6	.6	94.5
	Hispanic/Latino	4	2.3	2.4	97.0
	Mixed race	4	2.3	2.4	99.4
	Other	1	.6	.6	100.0
	Total	165	96.5	100.0	
Missing	999	6	3.5		
Total		171	100.0		
Race^a^					
Valid	Black	11	39.3	40.7	40.7
	White	16	57.1	59.3	100.0
	Total	27	96.4	100.0	
Missing	999	1	3.6		
Total		28	100.0		
Number of children in home^b^					
Valid	0	2	1.2	1.2	1.2
	1	47	27.5	27.6	28.8
	2	62	36.3	36.5	65.3
	3	35	20.5	20.6	85.9
	4	14	8.2	8.2	94.1
	5	3	1.8	1.8	95.9
	6	6	3.5	3.5	99.4
	7	1	.6	.6	100.0
	Total	170	99.4	100.0	
Missing	999	1	.6		
Total		171	100.0		
Number of children in the home^a^					
Valid	1	9	32.1	32.1	32.1
	2	9	32.1	32.1	64.3
	3	7	25.0	25.0	89.3
	4	1	3.6	3.6	92.9
	5	1	3.6	3.6	96.4
	6	1	3.6	3.6	100.0
	Total	28	100.0	100.0	
Age of youngest child in home^b^					
Valid	N/A	2	1.2	1.2	1.2
	<1 year	53	31.0	31.2	32.4
	1–5 years	63	36.8	37.1	69.4
	6–10 years	26	15.2	15.3	84.7
	11–15 years	15	8.8	8.8	93.5
	>15 years	11	6.4	6.5	100.0
	Total	170	99.4	100.0	
Missing	999	1	.6		
Total		171	100.0		
Age of youngest child in home^a^					
Valid	<1 year	9	32.1	32.1	32.1
	1–5 years	9	32.1	32.1	64.3
	6–10 years	5	17.9	17.9	82.1
	11–15 years	3	10.7	10.7	92.9
	>15 years	2	7.1	7.1	100.0
	Total	28	100.0	100.0	
Age of oldest child in home^b^					
Valid	N/A	50	29.2	29.4	29.4
	<1 year	2	1.2	1.2	30.6
	1–5 years	16	9.4	9.4	40.0
	6–10 years	34	19.9	20.0	60.0
	11–15 years	39	22.8	22.9	82.9
	>15 years	29	17.0	17.1	100.0
	Total	170	99.4	100.0	
Missing	999	1	.6		
Total		171	100.0		
Age of oldest child in home^a^					
Valid	N/A	9	32.1	32.1	32.1
	1–5 years	5	17.9	17.9	50.0
	6–10 years	3	10.7	10.7	60.7
	11–15 years	8	28.6	28.6	89.3
	>15 years	3	10.7	10.7	100.0
	Total	28	100.0	100.0	
Do you mind answering questions about guns?^b^					
Valid	No	118	69.0	70.2	70.2
	Yes	50	29.2	29.8	100.0
	Total	168	98.2	100.0	
Missing	999	3	1.8		
Total		171	100.0		
Do you mind answering questions about guns?^a^					
Valid	No	21	75.0	75.0	75.0
	Yes	7	25.0	25.0	100.0
	Total	28	100.0	100.0	
Have other parents asked you about guns?^b^					
Valid	No	164	95.9	95.9	95.9
	Yes	7	4.1	4.1	100.0
	Total	171	100.0	100.0	
Have other parents asked you about guns?^a^					
Valid	No	28	100.0	100.0	100.0
Have you asked other parents about guns?^b^					
Valid	No	125	73.1	74.4	74.4
	Yes	43	25.1	25.6	100.0
	Total	168	98.2	100.0	
Missing	999	3	1.8		
Total		171	100.0		
Do you ask other parents about guns?^a^					
Valid	No	17	60.7	60.7	60.7
	Yes	11	39.3	39.3	100.0
	Total	28	100.0	100.0	
Has any medical provider talked to you about gun safety?^b^					
Valid	No	136	79.5	79.5	79.5
	Yes	35	20.5	20.5	100.0
	Total	171	100.0	100.0	
Has any medical provider talked to you about gun safety?^a^					
Valid	No	21	75.0	75.0	75.0
	Yes	7	25.0	25.0	100.0
	Total	28	100.0	100.0	
Is your gun/ammunition locked?^b^					
Valid	Not Applicable	143	83.6	89.4	89.4
	No	13	7.6	8.1	97.5
	Yes	3	1.8	1.9	99.4
	Unsure	1	.6	.6	100.0
	Total	160	93.6	100.0	
Missing	999	11	6.4		
Total		171	100.0		
Is your gun/ammunition locked?^a^					
Valid	No	1	3.6	3.7	3.7
	Yes	26	92.9	96.3	100.0
	Total	27	96.4	100.0	
Missing	999	1	3.6		
Total		28	100.0		

^a^Have firearm in home? = Yes.

^b^Have firearm in home? = No.

## 4 Discussion

Findings from our study highlight a significant lack of screening of our pediatric patients both in the inpatient and outpatient settings, with the majority reporting that they had never been asked by a medical provider about firearm safety. In addition, three quarters of parents with a firearm in the home reported that they did not mind answering questions about firearms yet none had been asked by other parents about firearms. Thus, although firearm possession and safety is considered to be a sensitive topic, many parents are willing to discuss it with their health care providers and other parents.

The lack of discussion of firearm possession and safety is not limited to pediatric providers;100% of our respondents had never been asked by another parent about the presence of a firearm in their home when a child came over for a playdate. This illustrates an opportunity for pediatricians to increase education and encourage parents to speak to other parents about the presence of firearms within the household.

Hughes et al., completed a retrospective cross-sectional study of pediatric firearm injuries that resulted in an inpatient hospitalization at U.S. based hospitals. Their findings demonstrated that pediatric firearm injury circumstances and survival vary by race with white people being more likely to experience unintentional injury and suicide, while Black and Hispanic people are more likely to experience inflicted injury (53).Our lack of significant findings related to race demonstrate that providers do not address firearm safety regardless of parent race, even though we noted white parents were more likely than non- white parents to have a firearm in the home.

Our study showed no significant difference between parental age, number of children or ages of children in the home and provider counseling on firearm safety. Data shows that all pediatric patients are vulnerable to firearm injury and that the type of injury to which they are most susceptible depends on their age. Cheng et al., analyzed the national trauma data bank between 2013 and 2017 to evaluate firearm injuries in pediatric patients. There were a total of 20,223 incidences of pediatric firearm injury. Toddlers had a higher number of unintentional firearm discharges (*n *= 424, *p *< 0.001) compared to other discharge intents. However, for all other age groups, assaults were greater than unintentional firearm discharges (*p *< 0.001) (54). This highlights the need for universal screening with screening and counseling customized to the age of children in the home and other risk factors for firearm injuries.

Based on our data, the need for universal firearm screening is evident both in the inpatient and outpatient pediatric settings. There is a willingness of families to talk about firearm safety, however there is a missed opportunity to screen for firearms in the home and provide education on firearm safety with every patient encounter. Methods that may help to facilitate this include continuing to encourage primary care physicians to include firearm screening and firearm safety counseling within well child visits. Bhalakia et al., reviewed survey respondents within an outpatient group, where it was noted that only 32% of physicians report routine counseling. The most frequent barrier to providing education was insufficient time (63%), followed by unfamiliarity with firearms (26%) (55). Although there is limited data within the inpatient settings, Oddo et al. implemented a firearm screening tool within their tertiary pediatric hospital which demonstrated that inpatient firearm screening improved resident comfort when dealing with this sensitive topic. They noted firearm screening increased from 0.01% to 39% within a 25-month period through methods such as monthly reminders and nursing education on the importance of screening. Reported barriers included lack of time and feeling that it was not an appropriate setting for firearm screening. Possible proposed solutions to these barriers include adequate inpatient education to residents and nursing teams, and the incorporation of screening tools and education during patient intake and admissions (46). Firearm screening can also be expanded to the adult population, in both inpatient and outpatient settings.

There are several limitations to this study. We had a small sample size with 200 surveys. Of those 200, only 28 respondents had firearms in the home. This limits the generalizability of our results. Another limitation is missed opportunities when collecting survey responses due to several factors including parental presence on the pediatric floor and intensive care unit and isolation practices within the hospital. Future directions include creating an education module for providers to feel comfortable and more equipped when discussing firearm safety with families as well as incoperating firearm safety assessment upon patient intake in both inpatient and outpatient settings.

## 5 Conclusion

In the United States, firearm-related injuries are the leading cause of death among children and adolescents (1). Despite the risk that firearms pose to our patients, screening for the presence of firearms and discussions about firearm safety with patients and their families are limited. Our data highlights the willingness of families to discuss this important topic and a need for universal screening and education in both the inpatient and outpatient setting. Follow up studies are necessary to determine successful screening methods and safety education practices.

## Data Availability

The original contributions presented in the study are included in the article/Supplementary Material, further inquiries can be directed to the corresponding authors.
