# Supplementary material for: Firearm screening in pediatric patients
Source: Front Pediatr. 2024 Jun 21;12:1415612. doi: 10.3389/fped.2024.1415612 (PMC11228932; doi:10.3389/fped.2024.1415612)
Supplement: Supplementary file 1 [file Datasheet1.pdf]

Supplementary File 1: Pediatric Firearm Safety Survey

Pediatric Firearm Safety Survey

Please note: This survey is for research purposes. Your participation is completely voluntary. If you wish to participate, please complete the answers on this survey. For any concerns, or questions, you may contact Cortney Foster 110 S. Paca St. 8th Floor Baltimore, MD 21201, 410-328-6957. Thank you!

Age ___________

Sex ___________

Race ___________

Zip Code ___________

Number of children in your home ___________

Ages of Child(ren) in home ____

How did you enter the hospital?

☐ From the Emergency Department at UMMC

☐ Transferred from another hospital

Do you mind answering questions about guns? Yes No

Has any other parent asked you if you have a gun in your home before their child came over to play? Yes No

Do you ask other parents about guns in the homes where your children play? Yes No

Has any medical provider ever talked to you about gun safety? Yes No

Do you have a gun/ammunition in your home? Yes No

Is the gun/ammunition locked? Yes No

Thank you so much for your time and participation. Please review this handout for information on firearm safety.
