# Supplementary material for: Firearm screening in pediatric patients
Source: Front Pediatr. 2024 Jun 21;12:1415612. doi: 10.3389/fped.2024.1415612 (PMC11228932; doi:10.3389/fped.2024.1415612)
Supplement: Supplementary file 2 [file Datasheet2.pdf]

Supplementary File 2 : Handout provided at end of survey detailing dangers of firearms and safe storage practices.

Preventing Gun Injuries at Home

Some facts on firearm-related injuries

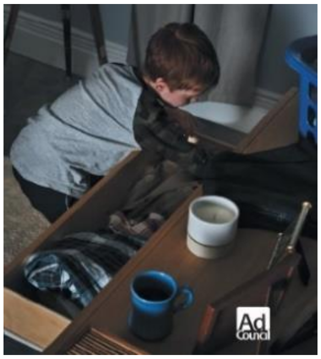

- Guns are the **2nd** leading cause of death in children
- Each day, **8** children and teens are unintentionally injured or killed due to an unlocked or unsupervised gun in the home
- **4.6 million** children live in homes with unlocked, loaded firearms
- **3 of 4** children report knowing where firearms are stored in their homes
- Children as young as **3 years** may be strong enough to pull the trigger on a handgun
- Access to guns at home increases the risk of suicide by **300%.**
- Locking all household firearms could reduce unintentional firearm fatalities and firearm suicide among youth by up to **32%.**

End Family Fire

- Family fire is a shooting involving an improperly stored or misused gun in the home that results in death or injury.

How can I end family fire?

- **ACT** responsibly by **researching** various gun storage options (e.g., cable locks, trigger locks, lock boxes, gun safes) to determine which is best for your family and **storing guns securely**.
- **ASK** before sending your child to a playdate, caretaker or relative's home: "**Is there an unlocked or loaded gun in the home?"**
  - Parent dropping off their kids for a playdate: "My kid is pretty curious, and our doctor recommended that I ask — is there an unlocked gun where my child will play?"
  - Teens taking a babysitting job: "Is there an unlocked and/or loaded gun in your home?"
- **TALK** with family, friends, and community about responsible gun ownership and teach them safe storage practices.
  - Remind your kids that if they come across a gun, they must stay away from it and tell you immediately.
- **LEARN** about specific safe storage options available in your area and take the time to familiarize yourself with the safe gun storage laws in your state.

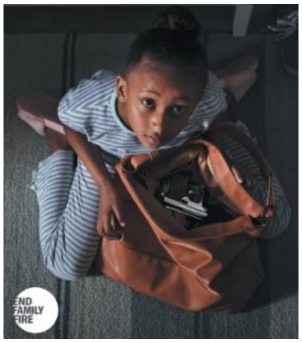

What is "safe storage"?

- Safe storage is keeping your guns **unloaded, locked** in a gun safe or box, **separate** from ammunition, and inaccessible to children at all times.

Steps for safe storage for all guns, every time:

- 1. Choose a safe or locking device that is appropriate for your firearms and their use
- 2. Before putting it in the safe or applying the locking device, ensure your firearm is unloaded.

- 3. Store the locked firearm in a location that is inaccessible to children and teens.
- 4. Store bullets separate from the gun in another locked box.
- 5. Lock the safe or locking device.

Resources

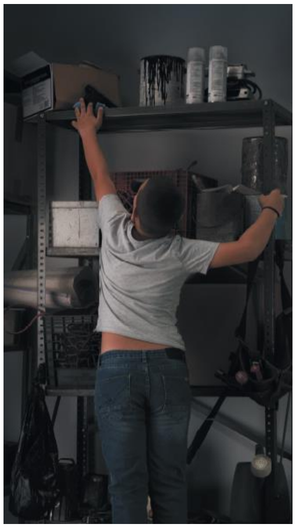

Where can I get safe storage devices?

- Gun safes and lock boxes are sold by a variety of retailers including Walmart, Amazon, and DICK'S Sporting Goods. Prices start at $20.
- Learn more at www.endfamilyfire.org

What can I do with a gun that I no longer want in my home?

- Firearms can be safely disposed of through gun surrender and gun buyback programs.
- Learn more at baltimorepolice.org

Are there laws in my state that require safe gun storage?

- Under Maryland law, a person "may not store or leave a loaded firearm in a location where the person knew or should have known that an unsupervised child would gain access to the firearm."
- Learn more about safe storage laws in other states at: http://lawcenter.giffords.org/gun-laws

For more information:

- Visit: www.bradycampaign.org and www.endfamilyfire.org

UNIVERSITY of MARYLAND
CHILDREN'S HOSPITAL

End Family Fire [Internet]. [cited 2024 Apr 3]. Together, We Can End Family Fire. Available from: https://www.endfamilyfire.org/
